# Genome-Wide Identification of the Soybean *AlkB* Homologue Gene Family and Functional Characterization of GmALKBH10Bs as RNA m^6^A Demethylases and Expression Patterns under Abiotic Stress

**DOI:** 10.3390/plants13172491

**Published:** 2024-09-05

**Authors:** Jie Zhao, Tengfeng Yang, Peng Liu, Huijie Liu, Hui Zhang, Sichao Guo, Xiaoye Liu, Xiaoguang Chen, Mingjia Chen

**Affiliations:** 1 College of Life Sciences, Nanjing Agricultural University, Nanjing 210095, China; 2 Department of Criminal Science and Technology, Nanjing Police University, Nanjing 210023, China; 3 School of Life and Environmental Sciences, Hangzhou Normal University, Hangzhou 311121, China

**Keywords:** AlkB homologue, m^6^A, RNA demethylation, soybean, abiotic stress

## Abstract

Soybean (*Glycine max* (L.) Merr) is one of the most important crops worldwide, but its yield is vulnerable to abiotic stresses. In Arabidopsis, the AlkB homologue (ALKBH) family genes plays a crucial role in plant development and stress response. However, the identification and functions of its homologous genes in soybean remain obscured. Here, we identified a total of 22 *ALKBH* genes in soybean and classified them into seven subfamilies according to phylogenetic analysis. Gene duplication events among the family members and gene structure, conserved domains, and motifs of all candidate genes were analyzed. By comparing the changes in the m^6^A levels on mRNA from hair roots between soybean seedlings harboring the empty vector and those harboring the GmALKBH10B protein, we demonstrated that all four GmALKBH10B proteins are *bona fide* m^6^A RNA demethylases in vivo. Subcellular localization and expression patterns of the GmALKBH10B revealed that they might be functionally redundant. Furthermore, an analysis of *cis*-elements coupled with gene expression data demonstrated that *GmALKBH10B* subfamily genes, including *GmALKBH10B1*, *GmALKBH10B2*, *GmALKBH10B3*, and *GmALKBH10B4*, are likely involved in the *cis*-elements’ response to various environmental stimuli. In summary, our study is the first to report the genome-wide identification of *GmALKBH* family genes in soybean and to determine the function of GmALKBH10B proteins as m^6^A RNA demethylases, providing insights into *GmALKBH10B* genes in response to abiotic stresses.

## 1. Introduction

N6-methyladenosine (m^6^A) is an abundant chemical modification in eukaryotic mRNA that is characterized by dynamic and reversible regulation, and it has a significant impact on RNA function in organisms. Similar to DNA methylation and histone methylation, m^6^A modification requires three types of enzymes including writer, eraser, and reader proteins that dynamically install, remove, and recognize RNA methylation information. In this system, RNA demethylation enzymes are considered to be crucial negative regulators of m^6^A modification. They are capable of recognizing and removing m^6^A modifications in RNA, which are involved in regulating the transcription, splicing, degradation, and translational regulatory processes of RNA [1,2]. Alpha-ketoglutarate-dependent dioxygenase (AlkB) homologues belong to a specific demethylation family. The members of this family were first identified as DNA repair proteins in *E. coli* [3]. Fat-mass- and obesity-associated proteins (FTOs) are a m^6^A demethylase found exclusively in mammals [4]. The discovery of the m^6^A demethylase FTO indicated that m^6^A modification was reversible. This was subsequently supported by the discovery of the m^6^A demethylase ALKBH5 (alkylated DNA repair protein AlkB homologue 5) [5]. Both proteins belong to the ALKBH family and are members of the Fe(II)/α-kg-dependent dioxygenase superfamily. However, no gene homologous to *FTO* was found in plants. Research on m^6^A demethylases in plants, therefore, has focused on candidate genes homologous to *ALKBH5*, such as *AtALKBH9B*, *AtALKBH10B*, and *SLALKBH2*. AtALKBH9B and AtALKBH10B are m^6^A demethylases in Arabidopsis that have been proven to remove m^6^A from single-stranded RNAs [6,7]. The m^6^A/A ratio increased in the *alkbh10b* mutants and decreased in the *ALKBH10B* overexpression line. In tomato, SlALKBH2 was demonstrated to be a m^6^A demethylase localized in the endoplasmic reticulum, which is crucial for fruit ripening [8].

Soybean (*Glycine max* (L.) Merr.) is a globally important food crop that provides protein for human consumption, livestock feed, and the biodiesel industry [9,10]. Additionally, its secondary metabolites, including anthocyanins and isoflavones, have potential for treating complex human diseases. However, soybean growth and development can be negatively impacted by several abiotic environmental stresses including global climatic extremes, drought, and salinity stress [11,12,13]. Many cellular processes are known to be involved in stress tolerance in soybean [14,15,16,17,18]. For instance, a recent study demonstrated that *pseudo-response regulator 3b (GmPRR3b)* negatively regulates the drought response by suppressing the expression of *abscisic acid-responsive element-binding factor 3* (*GmABF3*). Overexpressing *GmABF3* can significantly increase the ability to endure drought [14]. Additionally, RNA modifications, including m^6^A, play important roles in the response to abiotic stresses. After cadmium treatment, root growth was strongly suppressed in soybean plants. However, the presence of rhizobia can promote root growth and restore the growth performance induced by cadmium stress. These phenotypic alterations could be traced back to the reduction in m^6^A levels on specific genes involved in ROS homeostasis and calcium signaling [15]. In the presence of lead, the expression levels of many m^6^A-containing transcripts involved in lead uptake, transport, and accumulation are highly increased in soybean roots, hence enhancing their tolerance to lead accumulation [16]. m^6^A RNA modifications also play an important role in the response to light in soybean plants. The core genes in the light-signaling pathway, such as *suppressor of phA-105 1a* (*GmSPA1a*), *pseudo-response regulator 5e* (*GmPRR5e*), and *blue-light inhibitor of cryptochromes 2b* (*GmBIC2b*), exhibit changes in m^6^A levels on mRNA and in transcript abundance in response to light stimuli [17]. However, it remains unclear whether *ALKBH* family gene-mediated RNA demethylation in soybean is involved in the response to abiotic stress conditions. Therefore, identification and functional analysis of the role of the *GmALKBH* gene family in stress tolerance are urgently needed.

Here, we identified 22 *GmALKBH* candidate genes in soybean. The evolutionary relationship, chromosomal location, gene structure, and *cis*-element regulation of the *GmALKBH* family were analyzed. Among them, GmALKBH10B1, GmALKBH10B2, GmALKBH10B3, and GmALKBH10B4 were demonstrated to be m^6^A RNA demethylases using an in vivo enzymatic analysis. The tissue-specific expression and subcellular localization data suggest that the expression patterns of the four *GmALKBH10B* genes varied. In addition, the *GmALKBH10B* subfamily genes responded differently to various abiotic stresses. Hence, our study provides basic information on the *GmALKBH* gene family and the expression levels of *GmALKBH10Bs* upon abiotic stress treatment.

## 2. Results

### 2.1. Genome-Wide Identification of the GmALKBH Genes in Soybean

**Figure 1:**
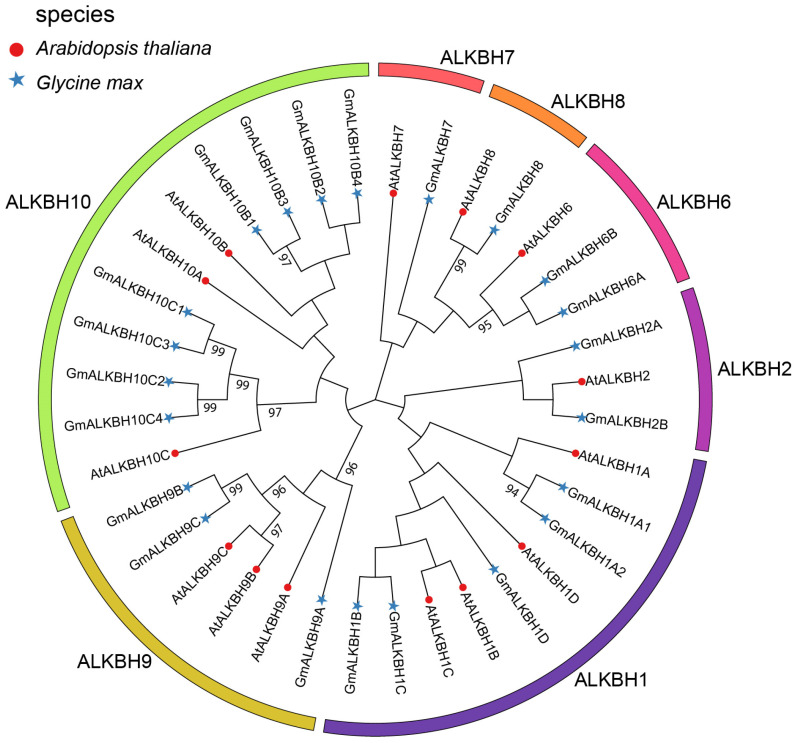
Phylogenetic analysis of ALKBH family proteins in *Arabidopsis thaliana* and *Glycine max.* An unrooted phylogenetic tree was generated via the neighbor-joining method via MEGA11 software; 1000 bootstraps.

To extensively identify the ALKBH proteins in soybean (*Glycine max* (L.) Merr.), a BLASTp analysis in the Phytozome V13 database was conducted using the amino acid sequences of the fourteen known Arabidopsis ALKBH family proteins [7,19]. As a result, twenty-four homologous proteins from the genome of *G. max* (Wm82.a4.v1) were obtained. Among them, twenty-two candidates (Table 1) were identified as GmALKBH family members employing the HMM module (Hidden Markov Model) of the AtALKBH protein sequences from Arabidopsis [20,21]. To further investigate the evolutionary relationship between *GmALKBH* genes and obtain a detailed classification of the *ALKBH* gene family in soybean, a neighbor-joining phylogenetic tree was constructed using MEGA 11 software. The tree was based on the multiple alignment of thirteen–six protein sequences from *Arabidopsis thaliana* (14 members) and *G. max* (22 members) (Supplementary Table S1), the *GmALKBH* genes were named according to their order on the chromosome in reference to the Arabidopsis *ALKBH* gene family. As shown in Figure 1, all of the *ALKBH* genes were divided into seven classes: ALKBH1, ALKBH2, ALKBH6, ALKBH7, ALKBH8, ALKBH9, and ALKBH10. Among them, *ALKBH10* subfamily had the largest number of genes, with eight *GmALKBH* genes and three *AtALKBH* genes. The four *GmALKBH10B* genes could be candidates for m^6^A RNA demethylases due to their close evolutionary relationship with *AtALKBH10B* (Figure 1). The *ALKBH1* subfamily has five *GmALKBH* genes and four *AtALKBH* genes. The GmALKBH1 proteins were predicted to be widely localized in the mitochondria, chloroplasts, and nucleus, suggesting that these proteins may have different functions. The remaining classes had a smaller number of genes, including *ALKBH2*, *ALKBH6*, *ALKBH7*, *ALKBH8*, and *ALKBH9*.

These GmALKBH proteins contained 123 to 683 amino acids, and their molecular weights varied from 14.32 to 73.46 kDa. The isoelectric points ranged from 5.71 to 9.86. The subcellular localization of these twenty-two GmALKBH proteins was analyzed using WoLF PSORT prediction. Among these proteins, nineteen GmALKBH proteins were located in the nucleus, one (GmALKBH1A1) in the chloroplast, one (GmALKBH1D) in the mitochondrion, and one (GmALKBH2B) in the extracellular region. Taken together, these results suggested that different GmALKBH proteins may have different biological functions.

### 2.2. Chromosomal Localization and Duplication Analysis of Soybean ALKBH Family Genes

**Figure 2:**
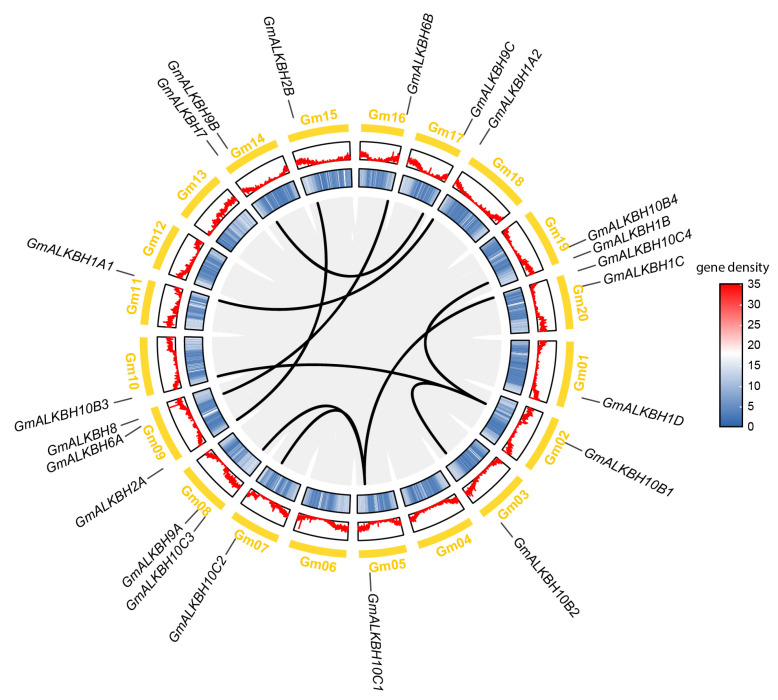
Genomic localization and collinearity analysis of the *ALKBH* family genes in soybean. Concentric circles, from outer to inner, show (1) soybean chromosomes, (2) GC content, (3) gene density, and (4) syntenic bloc; the ten black lines represent collinear pairs of *GmALKBH* family genes.

Next, we investigated the chromosomal distribution of the twenty-two *GmALKBH* genes across the soybean genome and the associated gene duplication events. As shown in Figure 2, all of the *GmALKBH* genes were randomly distributed on 16 of the 20 soybean chromosomes. Chromosome 9 has three *ALKBH* family members, chromosomes 8, 14, 19, and 20 each possess two, whereas chromosomes 1, 2, 3, 5, 7, 10, 11, 15, 16, 17, and 18 each contain only one gene. Intriguingly, eight of twenty-two *GmALKBH* family genes, including *GmALKBH1A1*, *GmALKBH1A2*, *GmALKBH1B*, *GmALKBH2A*, *GmALKBH6B*, *GmALKBH7*, *GmALKBH10B3*, and *GmALKBH10C4*, were exclusively distributed on the telomeric regions of the chromosome with low gene density. These results suggested that some *GmALKBH* genes may have contributed to the expansion and evolution of this gene family.

Various types of gene replication events, including whole genome duplication (WGD) and transposable duplication (TRD), widely occur in plant genomes, leading to the expansion of gene families [22]. Collinearity analysis of the *GmALKBH* family genes revealed that ten gene pairs (*GmALKBH1A1*/*GmALKBH1A2*, *GmALKBH2A*/*GmALKBH2B*, *GmALKBH6A*/*GmALKBH6B*, *GmALKBH9B*/*GmALKBH9C*, *GmALKBH10B1*/*GmALKBH10B2*, *GmALKBH10B1*/*GmALKBH10B3*, *GmALKBH10B1*/*GmALKBH10B4*, *GmALKBH10C1*/*GmALKBH10C2*, *GmALKBH10C1*/*GmALKBH10C3*, and *GmALKBH10C1*/*GmALKBH10C4*) arose from gene duplication events in soybean (Figure 2). Furthermore, a duplication event analysis using a publicly available database [23] suggested that many *ALKBH* family genes in soybean were the result of whole genome duplication, while others originated from transposable duplication (Table 2). Notably, no tandem duplication events occurred within the *GmALKBH* gene family. These results indicate that whole genome duplication events might be the reason for the expansion of the *GmALKBH* gene family.

### 2.3. Gene Structure and Conserved Motifs of the GmALKBH Family of Genes

**Figure 3:**
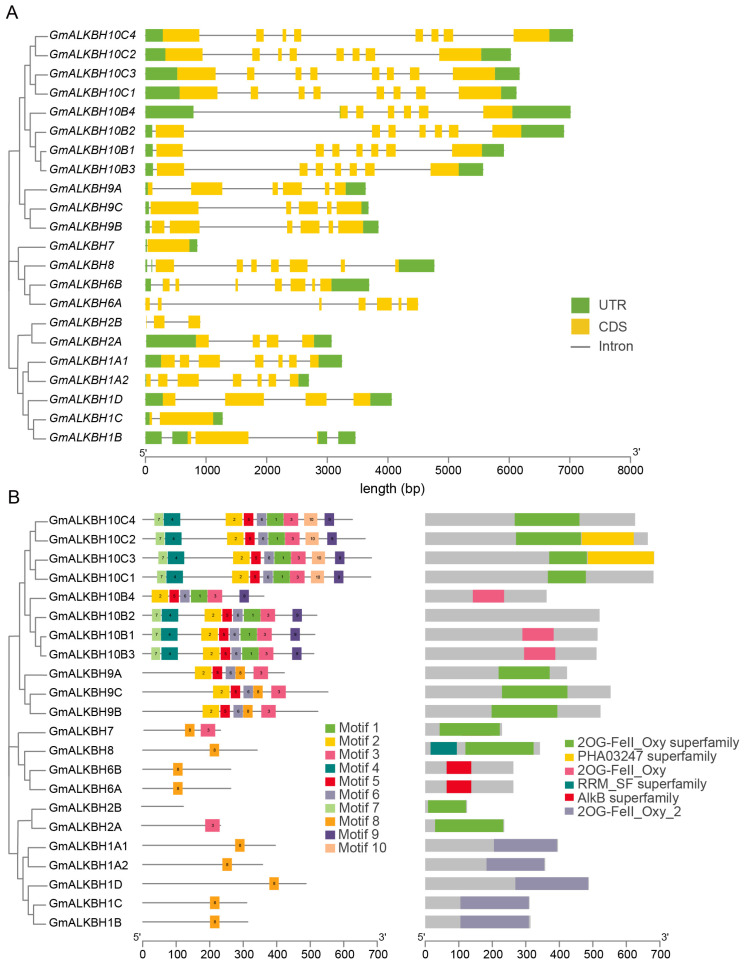
Gene structure, conserved domain, and motif analysis of the *GmALKBH* family genes. (**A**) Phylogenetic analysis and exon–intron structures of the *GmALKBH* family genes. The green boxes, yellow boxes, and gray lines represent untranslated regions (UTRs), coding sequences (CDSs), and introns, respectively. (**B**) Phylogenetic analysis and the conserved domain and motif distributions of the *GmALKBH* family genes.

We constructed a phylogenetic tree employing the neighbor-joining method to reconstruct the evolutionary relationships among the *GmALKBH* family genes (Figure 3), which was consistent with that of the phylogenetic analysis constructed using proteins from soybean and Arabidopsis (Figure 1). To investigate the genetic structural diversity among the *GmALKBH* family genes, an exon–intron distribution analysis was performed. Notably, the members from each subfamily possessed similar gene structures. For example, four members (*GmALKBH10C1*, *GmALKBH10C2*, *GmALKBH10C3*, and *GmALKBH10C4*) of the *GmALKBH10C* subfamily had the most exons (8 exons), while *GmALKBH7* contained only one exon (Figure 3A). Generally, the candidate genes from different subfamilies were complex and diverse in structure, suggesting that the *GmALKBH* genes retained their original function and might also expand towards evolving new functions.

We also employed a MEME program to identify the conserved motifs present within the *GmALKBH* family genes. In total, ten different and highly conserved motifs were observed (Figure 3B and Supplementary Table S2). Motif 2, motif 3, motif 5, and motif 6 were unique to the *GmALKBH9* and *GmALKBH10* subfamilies, suggesting that they may be important for demethylase activity. However, no conserved motif was detected in *GmALKBH2B*, possibly because this gene has a new function. Most genes, except *GmALKBH10B2,* possessed conserved domains, including the 2OG-Fe(II)_Oxy, 2OG-Fe(II)_Oxy2, 2OG-Fe(II)_Oxy superfamily, or AlkB superfamily. The homologous proteins of each subfamily share identical conserved motifs, suggesting that they may be functionally redundant.

### 2.4. Cis-Element Analysis of the GmALKBH Family of Genes

**Figure 4:**
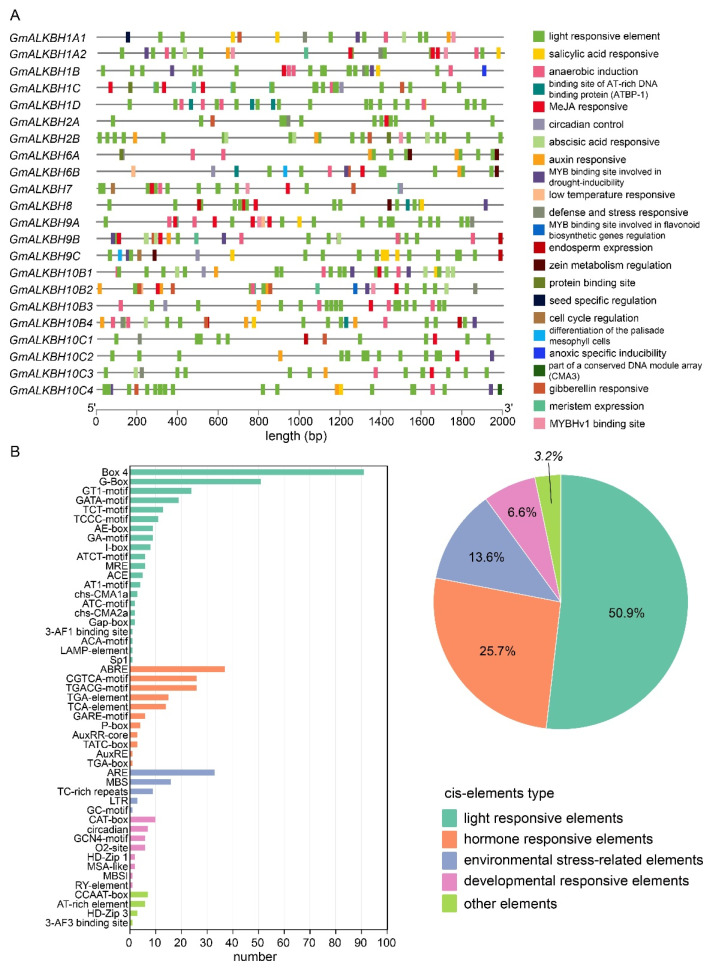
Prediction of *cis*-elements in the *GmALKBH* family gene promoter. (**A**) *Cis*-element distribution of the *GmALKBH* family gene promoter. Different *cis*-elements are represented by different colors. (**B**) The classification of the *cis*-elements and the proportions of different types of *cis*-elements. The 519 *cis*-elements were divided into five groups, including 269 light-responsive elements, 136 hormone-responsive elements, 62 environmental stress-related elements, 35 developmental responsive elements, and 17 other elements.

To better understand the transcriptional regulatory activities of *GmALKBH* genes, we predicted the *cis*-elements within 2000 bp promoter regions of *GmALKBH* genes by employing the PlantCARE service website analysis (PlantCARE, a database of plant promoters and their cis-acting regulatory elements (ugent.be)). In total, *cis*-elements involved in 23 functional categories were identified in *GmALKBH* genes (Figure 4A), which could be classified into five groups: light responsive, hormone responsive, environmental stress related, developmental response, and other elements (Figure 4B, right panel). Interestingly, light-responsive elements, which accounted for 50.9% of the total *cis*-elements (Figure 4), were identified in all of the promoters of the *GmALKBH* family genes (Figure 4A). Among the hormone-responsive elements, those involved in the abscisic acid (ABA), methyl jasmonate (MeJA), auxin, and gibberellin response were highly abundant. The most frequent environmental stress-related element was anaerobic induction. In addition, *cis*-elements related to the developmental response and others were also identified (Figure 4). The high abundance of light-, MeJA-, auxin-, and gibberellin-responsive elements identified in the promoters of the *GmALKBH* family genes suggested that the expression levels of these genes were likely influenced by light and several phytohormones, which in turn may affect the development and environmental stimulus responses of soybean.

### 2.5. GmALKBH10Bs Are RNA N6-Methyladenosine Demethylases in Soybean

**Figure 5:**
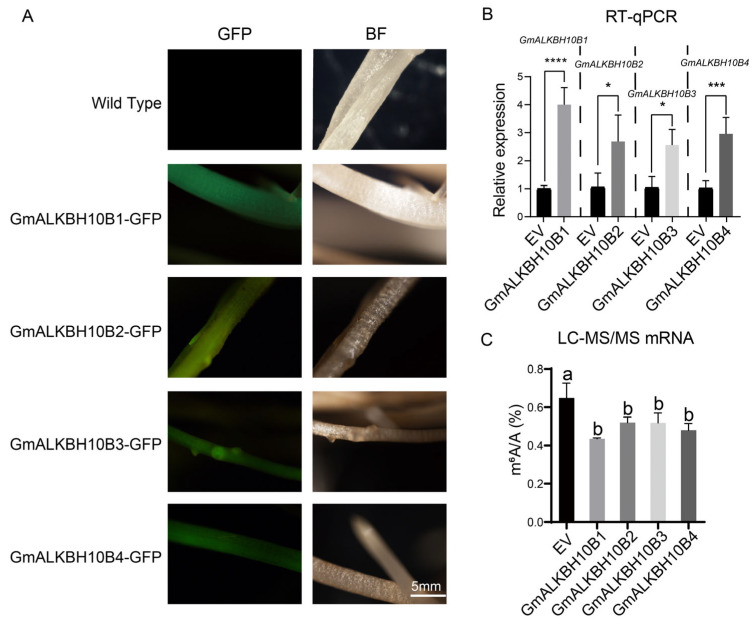
Demethylase activities of GmALKBH10B proteins. (**A**) Overexpression of *GmALKBH10B* genes in the hair roots of soybean plants. Green from GFP. Bar, 5 mm. BF, brightfield. (**B**) Relative *GmALKBH10B* genes’ expression in control (EV) and positive hairy roots (*GmALKBH10B1*, *GmALKBH10B2*, *GmALKBH10B3*, *GmALKBH10B4*) determined via real-time quantitative PCR (RT–qPCR). The error bars represent the SDs (n = 3, * *p ≤* 0.05; *** *p ≤* 0.001; **** *p ≤* 0.0001). (**C**) The m^6^A level in mRNA in control (EV) and positive hairy roots (*GmALKBH10B1*, *GmALKBH10B2*, *GmALKBH10B3*, *GmALKBH10B4*) determined via liquid chromatography–tandem mass spectrometry (LC–MS/MS). The error bars represent the SDs (n = 3). Different letters indicate significant differences at *p* < 0.05.

In Arabidopsis, AtALKBH10B efficiently demethylates m^6^A on RNA [7]. To test whether GmALKBH10B1, GmALKBH10B2, GmALKBH10B3, and GmALKBH10B4, orthologues of AtALKBH10B in soybean, are RNA m^6^A demethylases, we independently overexpressed these four *GmALKBH10B* genes recombinantly in soybean plants (Figure 5A,B), employing a well-established hair roots expression system [24,25,26,27], and detected the m^6^A level on mRNA from root tissue via liquid chromatography–tandem mass spectrometry (LC–MS/MS). Remarkably, the results revealed a significant decrease in m^6^A abundance upon overexpression of the four *GmALKBH10Bs* compared with that in the control (Figure 5C). These experimental results strongly demonstrate that all four GmALKBH10B proteins possess demethylase activities, showing their ability to remove mRNA m^6^A modifications (Figure 5).

### 2.6. Spatiotemporal Expression and Subcellular Localization of GmALKBH10B Subfamily Members

By integrating data from the SoyOmic website (https://ngdc.cncb.ac.cn/soyomics/index, accessed on 5 July 2023), we next investigated the spatial expression patterns of all of the *GmALKBH10B* genes across various tissues from *G.max*, including seeds, roots, lateral roots, nodules, stems, leaves, and flowers. The results show distinct expression patterns among these four genes across different tissue types (Supplementary Figure S1). Intriguingly, we observed that *GmALKBH10B3* was significantly up-regulated in flowers and lateral roots, while *GmALKBH10B2* was relatively increased in nodules and stems, demonstrating its potential involvement in different developmental processes in soybean. Furthermore, a protein sequence alignment (Supplementary Figure S2) and motif analysis (Figure 3B) of the GmALKBH10B subfamily revealed high similarity and consistent motif distribution in conserved regions, with no significant differences noted. In contrast, all four genes exhibited low expression levels in seeds, suggesting that GmALKBH10Bs-mediated RNA m^6^A demethylation might not be required for seed development.

**Figure 6:**
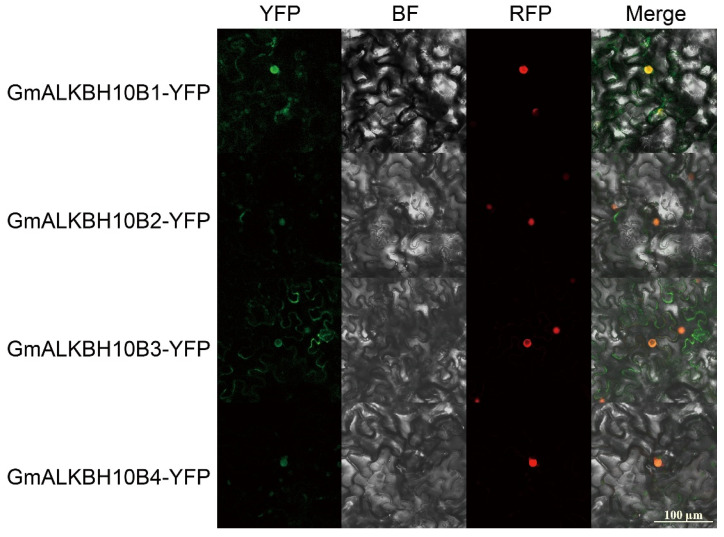
Subcellular localization of GmALKBH10B proteins in *Nicotiana benthamiana* leaves. Green represents YFP, red represents RFP, and yellow represents the overlap of green and red. Bar, 100 μM. BF, brightfield.

In addition, we used a publicly available database (WoLF PSORT, https://wolfpsort.hgc.jp/, accessed on 29 July 2023) to predict the subcellular localization of GmALKBH10B proteins and other members of the GmALKBH10B family. The results show that GmALKBH10B subfamily members, like most other GmALKBH proteins, were all localized in the nucleus (Table 1). To validate this prediction, C-terminal yellow fluorescent protein (YFP) was fused with the full-length amino acid sequences of four GmALKBH10B members and then independently transiently co-expressed with the nuclear marker Histone 2B fused with mCherry (H2B-RFP) in *Nicotiana benthamiana* leaves. The results reveal that all four proteins predominantly accumulated in the nucleus (Figure 6), which is in line with our prediction.

### 2.7. Expression Patterns of GmALKBH10B Genes in Response to Abiotic Stress

**Figure 7:**
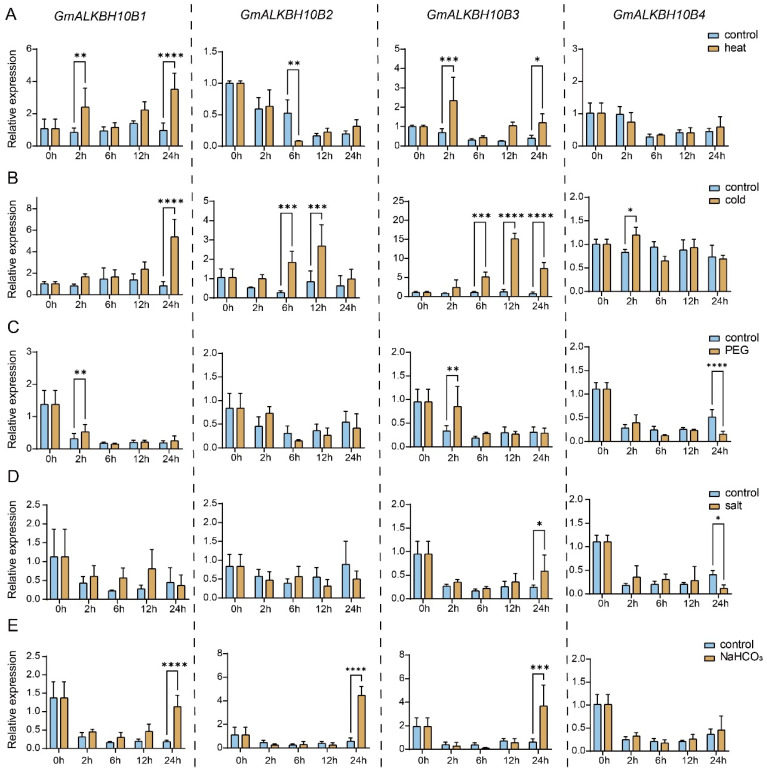
Relative expression quantified using real-time quantitative PCR (RT–qPCR) of *GmALKBH10B* genes in leaves under heat (**A**), cold (**B**), polyethylene glycol (PEG, (**C**)), salt (**D**), and alkalinity (NaHCO_3_, (**E**)) abiotic stresses. All of the results shown were normalized to *GmF-BOX* (*Glyma.12G051100*) expression as an internal control. The error bars represent the SDs (*n =* 3, * *p ≤* 0.05; ** *p ≤* 0.01; *** *p ≤* 0.001; **** *p ≤* 0.0001).

The results of the *cis*-element analyses suggest that the expressions of *GmALKBH10B* subfamily genes were likely regulated by different abiotic stresses. We therefore analyzed the expression levels of *GmALKBH10B1*, *GmALKBH10B2*, *GmALKBH10B3*, and *GmALKBH10B4* in the leaves of 2-week-old soybean plants grown under normal conditions or different stress conditions, including heat, cold, drought, salinity, and alkalinity, over 24 h. Generally, abiotic stress treatments significantly altered the expression patterns of four *GmALKBH10B* genes (Figure 7). However, each gene showed a different response to those stress conditions. For instance, the expression of *GmALKBH10B2* and *GmALKBH10B3* increased after 6 h of cold treatment, whereas the expression of *GmALKBH10B1* and *GmALKBH10B4* increased after only 24 h and 2 h, respectively, compared with that of the control. In addition, the expression levels of *GmALKBH10B1*, *GmALKBH10B2*, and *GmALKBH10B3* remained unchanged until 24 h after alkalinity treatment. In the drought treatment group, the expression of both *GmALKBH10B1* and *GmALKBH10B3* significantly increased after 2 h, whereas *GmALKBH10B4* expression decreased after 24 h compared with that in the control group (Figure 7). Taken together, these results revealed that GmALKBH10Bs-mediated RNA m6A demethylation likely participates in the abiotic stress response in soybean.

## 3. Discussion

RNA demethylation mediated by ALKBH family proteins plays a crucial role in the regulation of plant growth, development, and abiotic stress responses [19,28,29,30]. However, the molecular mechanism of the *ALKBH* genes, especially in legume plants, has remained unclear. In this study, we identified 22 GmALKBH proteins in soybean via the alignment of homologous amino acid sequences of ALKBH in Arabidopsis (Figure 1). These homologous ALKBHs were assigned to seven subfamilies, namely ALKBH1, ALKBH2, ALKBH6, ALKBH7, ALKBH8, ALKBH9, and ALKBH10, which was in line with previous analyses in tomato and Populus [28,31]. Protein function is frequently conserved between paralogs [32]. Similar to the function of AtALKBH10B in Arabidopsis [7], we demonstrated the m^6^A demethylase activities of the four GmALKBH10B proteins by an in vivo assay employing a transient expression system in the hair roots of soybean plants (Figure 5). Simultaneously, we performed a subcellular localization analysis of four GmALKBH10B proteins, which indicated that they were all localized in the nucleus (Figure 6), in line with our prediction (Table 1).

Unlike the known *AtALKBH* family genes, more *GmALKBHs* were identified from *G. max*. Gene duplications are considered to be one of the main driving forces of genetic evolution in the paleopolyploid soybean genome [33]. Different gene replication events, such as tandem, fragment duplication, and transposition events, frequently occur in the soybean genome, resulting in the expansion of gene families [22]. Ten gene pairs from the *GmALKBH* family were involved in gene duplication events (Figure 2). Many whole genome duplications and few transposable duplication events, but not tandem duplication events, contributed to the expansion of the *GmALKBH* gene family (Table 2).

In addition, the gene structures of all *GmALKBH* family genes were analyzed, with similar structures and conserved motifs present among the members of each subfamily, suggesting a shared evolutionary origin and potential functional similarity among the subfamily members. However, variations in gene structure could still be observed within the same subfamily, which might arise from spontaneous mutations or transposed duplication-induced genetic information loss occurring in the soybean genome (Table 2). Such changes in gene structure encompass alterations or losses of genetic content within intron–exon regions and potentially influence gene function (Figure 3).

In plants, RNA methylation (m^6^A) is involved in the response to abiotic stresses [17,19,34,35,36]. However, whether *GmALKBH10Bs,* an RNA N6-methyladenosine demethylase-encoding gene, respond to abiotic stress conditions remains unknown. In our study, *cis*-elements on 2000 bp promoter sequences of all *GmALKBH* family genes were analyzed, and the results suggested that these genes might respond to light, phytohormones, and environmental stresses (Figure 4). In particular, ABA response elements (ABREs) were highly enriched in the promoter regions of many *GmALKBH* family genes, including *GmALKBH10B1*, *GmALKBH10B2*, and *GmALKBH10B4*. These data suggest that several *ALKBH* genes might be the targets of ABA signaling regulation in soybean. ABA signaling is well known to play an important role in the response to various stress conditions, such as drought, salinity, and cold stresses [33]. Hence, *GmALKBH* genes might be involved in ABA-mediated stress responses. This hypothesis has been confirmed in Arabidopsis and tomato [30,37,38]. In Arabidopsis, *alkbh10b* mutants are sensitive to ABA, osmotic, and salt stress during seed germination [30]. Similarly, the knock-out mutant of *SlALKBH10B* increased sensitivity to ABA treatment and up-regulation of gene expression related to ABA synthesis and response [38]. In addition, our qPCR analysis revealed that *GmALKB10B* genes were likely involved in cold, drought, and alkalinity stimuli.

In conclusion, our study identifies twenty-two *GmALKBH* family genes in soybean and demonstrates the enzymatic activities of four GmALKBH10Bs, providing new insights into their evolutionary process, genetic diversity, and potential functions in response to abiotic stress. These results serve as the foundation for future research on elucidating the molecular mechanisms underlying GmALKBH-mediated RNA demethylation in response to abiotic stress.

## 4. Method

### 4.1. Plant Materials and Abiotic Treatment

Soybean (*Glycine max*, Williams 82) seeds were cultivated on full-nutrient soil in the greenhouse under the following conditions: 16/8 h day/night cycle, 25 °C/24 °C day/night temperature, 70% humidity, and 100 μmol m^−2^ s^−1^ light intensity. For this study, 13-day-old plants were used as research materials, and different abiotic treatments were applied. For the cold and heat treatments, soybean seedlings were exposed to temperatures of 8 °C or 42 °C for a duration of 24 h. To induce drought stress, the seedlings were transferred to a Hoagland liquid culture supplemented with 20% polyethylene glycol (PEG, Sangon Biotech, Shanghai, China) and incubated for one day. For salinity and alkalinity stresses, the plants were grown in Hoagland liquid culture containing 150 mM NaCl (Sangon Biotech, Shanghai, China) or 100 mM NaHCO_3_ (Sangon Biotech, Shanghai, China), respectively, also for one day. Leaves from single plants were collected at 0 h, 2 h, 6 h, 12 h, and 24 h after treatment. All samples were immediately frozen in liquid nitrogen after harvesting, and were stored at −80 °C until use.

### 4.2. Identification of GmALKBH Gene Family Members and Construction of the Phylogenetic Tree of the ALKBH Family

A BLASTp analysis and a Hidden Markov Model (HMM) based on published AtALKBH protein sequences from Arabidopsis were constructed to search for soybean genomes (*Glycine max* Wm82.a4.v1) in Phytozome v13 database (https://phytozome-next.jgi.doe.gov/, accessed on 24 June 2023). After removing repeated sequences, a total of 22 putative candidates, including gene IDs and full-length amino acid sequences, were obtained. The CD-Search and InterPro programs were used to detect and confirm the presence of conserved domains in each identified sequence. The molecular weight (MW) and isoelectric point (pI) were predicted using the ExPASy tool.

The protein sequences of the *ALKBH* gene family from *A. thaliana* and *G. max* were used to construct a phylogenetic tree using MEGA11 [39] with the neighbor-joining (NJ) method and 1000 bootstrap replications. Thereafter, the phylogenetic tree was visualized using ChiPlot (https://www.chiplot.online/, accessed on 15 August 2023) [40].

### 4.3. Chromosomal Location and Duplication Analysis of the GmALKBH Gene Family

The physical position of each *GmALKBH* gene in the *Glycine max* Wm82.a4.v1 genome annotation was used to determine its chromosomal localization. The GFF data, including length information for all soybean chromosomes, from Phytozome were used to extract the positional information and gene density profiles of the *GmALKBH* genes on the chromosomes. The three datasets were then uploaded to TBtools for the analysis of *GmALKBH* genes’ chromosomal positions. To investigate the collinearity relationships among *GmALKBH* genes, the One Step MCScanX-Super Fast program integrated into TBtools was employed. All *GmALKBH* genes were categorized based on whole genome duplication (WGD) and transposable duplication (TRD) [41].

### 4.4. Gene Structure, Conserved Domain, Motif Analysis, and Cis-Element Analysis of GmALKBH Genes in Soybean

Gene structure analysis was performed based on GFF data extracted from Phytozome (https://phytozome-next.jgi.doe.gov/, accessed on 15 August 2023) to determine UTRs, exons, and introns. Conserved motifs of GmALKBH proteins were identified using the online service MEME (https:/meme-suite.org/meme/, accessed on 17 August 2023) with a maximum of ten motifs. The conserved domains of the GmALKBH protein sequences were identified using the Batch CD-Search program. For the *cis*-element analysis, the promoter region for each *GmALKBH* gene was defined as the 2000 base pairs upstream of the genomic sequence from the translation initiation site. The PlantCARE software (https://bioinformatics.psb.ugent.be/webtools/plantcare/html/, accessed on 17 August 2022) was used for prediction. Four types of analysis were visualized using the TBtools software (v2.121).

### 4.5. RNA Isolation, cDNA Synthesis and Quantitative Real-Time PCR (qRT–PCR)

Total RNA was isolated from 14-day-old soybean plants, and cDNA was prepared according to previous studies [42,43,44]. To analyze mRNA abundance, real-time quantitative PCR (RT–qPCR) was performed with QuantStudio 1 (Thermo Fisher Scientific, Waltham, MA, USA) using Hieff qPCR SYBR Green Master Mix (Yeasen Biotechnology, Shanghai, China) according to the previous description [43]. The transcript abundances of *GmALKBH10B1*, *GmALKBH10B2*, *GmALKBH10B3*, and *GmALKBH10B4* were analyzed by employing the primer pairs Cp641/Cp642, Cp643/Cp644, Cp645/Cp646, and Cp639/Cp640 (Supplementary Table S3), respectively. *GmF-BOX* (*Glyma.12G051100*) was amplified from cDNA with the primer pair Cp363/Cp364 as an internal reference gene. The calculation was based on the 2^−ΔΔCT^ method [45]. All primer sequences are detailed in Supplementary Table S3.

### 4.6. Cloning and Vector Construction

The following primers were used for cloning: *GmALKBH10B1* (*Glyma.02G149900*), Cp996 and Cp998; *GmALKBH10B2* (*Glyma.03G149900*), Cp997 and Cp999; *GmALKBH10B3* (*Glyma.10G023900*), Cp996 and Cp1000; and *GmALKBH10B4* (*Glyma.19G152900*), Cp998 and Cp1001 (Supplementary Table S3). The pXCS-YFP (V36) [46] was digested with SmaI (New England Biolabs, Beverly, MA, USA). *GmALKBH10B1*, *GmALKBH10B2*, *GmALKBH10B3*, and *GmALKBH10B4* were subsequently inserted into V36 through Hieff Clone^®^ Universal One Step Cloning Kit (Yeasen Biotechnology, Shanghai, China) for subcellular localization analysis. All primer sequences are detailed in Supplementary Table S3.

### 4.7. Soybean Hairy Root Transformation

A modified soybean hairy-root transformation method was utilized based on a previous study [24,25,26,27] to generate transgenic soybean lines. Transformed *Agrobacterium rhizogenes* strain K599 cells were streaked onto the surface of a TY medium containing the appropriate antibiotic and incubated at 28 °C until the appropriate bacterial growth state and cell density were reached. The soybean plants, which were grown to the state of spreading true leaves, were used for the transformation experiments. The plants were cut diagonally at 45° at 0.5–1 cm below the soybean cotyledons, and the bacterial chunks were evenly spread on the wounds using an aseptic loop. After a day of recovery in the dark, the plants were placed on dampened vermiculite and cultivated under normal growth conditions. After 15 days, the hairy roots were analyzed using an MVX10 microscope (Olympus Corporation, Tokyo, Japan), and the positive hairy roots displaying GFP fluorescence signals were collected and frozen at −80 °C for subsequent experiments.

### 4.8. Subcellular Localization and Tissue-Specific Expression of GmALKBH10B Genes

GmALKBH10B-eYFP was transiently co-expressed with the nuclear marker protein H2B-RFP (RFP fused to histone 2B) [47] in *Nicotiana benthamiana* leaves for 5 days. The samples were analyzed using a ZEISS LSM 980 instrument with an Airyscan2 microscope equipped with an HC PLAPO CS2 40 × 1.0 water immersion objective (ZEISS Microsystems) according to a previous description [48]. The data on the tissue-specific expression of *GmALKBH10B*, including flowers, lateral roots, leaves, nodules, roots, seeds, and stems, were downloaded from the transcriptome module of the Soyomics database (https://ngdc.cncb.ac.cn/soyomics/index, accessed on 15 August 2022) and visualized using Chiplot (https://www.chiplot.online/, accessed on 15 August 2022).

### 4.9. Quantitative Analysis with LC–MS/MS

Approximately 800 ng of mRNA was fully digested into single nucleosides as described previously [49]. After incubation at 37 °C for 10 h, the sample was filtered through an ultrafiltration tube (3 kDa cut-off; Pall). Two-microliter aliquots were analyzed by an Agilent 1290 HPLC system coupled with a Sciex 6500 QTRAP mass spectrometer (AB Sciex, Frisco, TX, USA). The following mass transitions were monitored: *m*/*z* 268.1 to 136 (A, adenosine) and *m*/*z* 282.12 to 150 (m^6^A, N6-methyladenosine). Standard solutions of A (1, 5, 25 50, 100, 200, 400, 2000, and 10 000 ng/mL, Sigma-Aldrich, St. Louis, MO, USA) and m^6^A (0.1, 0.5, 2.5, 5, 10, 20, 40, 200, and 1000 ng/mL, Sigma-Aldrich, St. Louis, MO, USA) were used for quantification. The ratio of m6A to A was calculated based on the calibrated concentrations.

### 4.10. Statistics

Statistical analysis was performed with GraphPad Prism 9.5.1 software. The statistical methods and sample sizes used are described in the figure legends. All of the replicates were biological replicates or experimental replicates.

## Figures and Tables

**Table 1:** Protein characteristics of the predicted *ALKBH* candidate genes in *Glycine max*.

Gene Name	Gene ID (Phytozome)	Amino Acid Length	Isoelectric Point	Molecular Weight (kDa)	Subcellular Localization Prediction
*GmALKBH1A1*	Glyma.11G250800	396	7.13	45.05	chloroplast
*GmALKBH1A2*	Glyma.18G006200	358	5.89	40.53	nucleus
*GmALKBH1B*	Glyma.19G263000	314	9.33	34.62	nucleus
*GmALKBH1C*	Glyma.20G056000	311	8.72	34.37	nucleus
*GmALKBH1D*	Glyma.01G129600	488	8.94	54.13	mitochondrion
*GmALKBH2A*	Glyma.09G014800	236	9.14	27.12	nucleus
*GmALKBH2B*	Glyma.15G120500	125	9.86	14.32	extracellular space
*GmALKBH6A*	Glyma.09G156400	263	6.26	30.03	nucleus
*GmALKBH6B*	Glyma.16G207100	263	6.20	29.79	nucleus
*GmALKBH7*	Glyma.14G026500	229	5.91	25.75	nucleus
*GmALKBH8*	Glyma.09G217100	342	6.83	38.34	nucleus
*GmALKBH9A*	Glyma.08G186500	423	6.29	48.00	nucleus
*GmALKBH9B*	Glyma.14G106000	523	6.22	58.58	nucleus
*GmALKBH9C*	Glyma.17G220300	553	7.60	62.19	nucleus
*GmALKBH10B1*	Glyma.02G149900	514	5.95	56.37	nucleus
*GmALKBH10B2*	Glyma.03G149900	520	5.88	57.46	nucleus
*GmALKBH10B3*	Glyma.10G023900	511	5.71	56.08	nucleus
*GmALKBH10B4*	Glyma.19G152900	362	8.66	40.24	nucleus
*GmALKBH10C1*	Glyma.05G138600	681	6.79	73.69	nucleus
*GmALKBH10C2*	Glyma.07G175300	664	6.61	71.74	nucleus
*GmALKBH10C3*	Glyma.08G093800	683	7.12	73.46	nucleus
*GmALKBH10C4*	Glyma.20G012100	626	6.73	68.04	nucleus

**Table 2:** Gene duplication identified in the *GmALKBH* gene family.

Duplicated 1	Duplication 2	Duplication Event
*GmALKBH1A1*	*GmALKBH1A2*	whole genome duplication
*GmALKBH1B*	*GmALKBH1D*	transposed duplication
*GmALKBH1C*	*GmALKBH1D*	transposed duplication
*GmALKBH2A*	*GmALKBH2B*	whole genome duplication
*GmALKBH6A*	*GmALKBH6B*	whole genome duplication
*GmALKBH9A*	*GmALKBH9B*	transposed duplication
*GmALKBH9B*	*GmALKBH9C*	whole genome duplication
*GmALKBH10B1*	*GmALKBH10B2*	whole genome duplication
*GmALKBH10B1*	*GmALKBH10B3*	whole genome duplication
*GmALKBH10B2*	*GmALKBH10B3*	whole genome duplication
*GmALKBH10B2*	*GmALKBH10B4*	whole genome duplication
*GmALKBH10C1*	*GmALKBH10C2*	whole genome duplication
*GmALKBH10C1*	*GmALKBH10C3*	whole genome duplication
*GmALKBH10C1*	*GmALKBH10C4*	whole genome duplication
*GmALKBH10C2*	*GmALKBH10C3*	whole genome duplication
*GmALKBH10C2*	*GmALKBH10C4*	whole genome duplication
*GmALKBH10C3*	*GmALKBH10C4*	whole genome duplication

## Data Availability

Data are contained within the article and Supplementary Materials.

## Supplementary Materials

The following supporting information can be downloaded at: https://www.mdpi.com/article/10.3390/plants13172491/s1, Supplementary Figure S1: Tissue-Specific Expression of *GmALKBH10B* Genes, Supplementary Figure S2: GmALKBH10B subfamily proteins sequence alignment, Supplementary Table S1: Predicted *ALKBH* family genes from *Arabidopsis thaliana* and *Glycine max*, Supplementary Table S2: Predicted conserved motif within the *GmALKBH* family genes in *G. max*, Supplementary Table S3: Primers used in this study.

## References

[1] Ries R.J., Zaccara S., Klein P., Olarerin-George A., Namkoong S., Pickering B.F., Patil D.P., Kwak H., Lee J.H., Jaffrey S.R. (2019). m^6^A Enhances the Phase Separation Potential of mRNA. Nature.

[2] Chen M., Urs M.J., Sánchez-González I., Olayioye M.A., Herde M., Witte C.-P. (2018). m^6^A RNA Degradation Products Are Catabolized by an Evolutionarily Conserved N6-Methyl-AMP Deaminase in Plant and Mammalian Cells. Plant Cell.

[3] Kataoka H., Yamamoto Y., Sekiguchi M. (1983). A New Gene (alkB) of *Escherichia coli* That Controls Sensitivity to Methyl Methane Sulfonate. J. Bacteriol..

[4] Jia G., Fu Y., Zhao X., Dai Q., Zheng G., Yang Y., Yi C., Lindahl T., Pan T., Yang Y.-G. (2011). N6-Methyladenosine in Nuclear RNA Is a Major Substrate of the Obesity-Associated FTO. Nat. Chem. Biol..

[5] Zheng G., Dahl J.A., Niu Y., Fedorcsak P., Huang C.-M., Li C.J., Vågbø C.B., Shi Y., Wang W.-L., Song S.-H. (2013). ALKBH5 Is a Mammalian RNA Demethylase That Impacts RNA Metabolism and Mouse Fertility. Mol. Cell.

[6] Martínez-Pérez M., Aparicio F., López-Gresa M.P., Bellés J.M., Sánchez-Navarro J.A., Pallás V. (2017). Arabidopsis m^6^A Demethylase Activity Modulates Viral Infection of a Plant Virus and the m^6^A Abundance in Its Genomic RNAs. Proc. Natl. Acad. Sci. USA.

[7] Duan H.-C., Wei L.-H., Zhang C., Wang Y., Chen L., Lu Z., Chen P.R., He C., Jia G. (2017). ALKBH10B Is an RNA N6-Methyladenosine Demethylase Affecting Arabidopsis Floral Transition. Plant Cell.

[8] Zhou L., Tian S., Qin G. (2019). RNA Methylomes Reveal the m6A-Mediated Regulation of DNA Demethylase Gene SlDML2 in Tomato Fruit Ripening. Genome Biol..

[9] Wilcox J.R., Shibles R.M., Harper J.E., Wilson R.F., Shoemaker R.C. (2016). World Distribution and Trade of Soybean. Agronomy Monographs.

[10] Koberg M., Abu-Much R., Gedanken A. (2011). Optimization of Bio-Diesel Production from Soybean and Wastes of Cooked Oil: Combining Dielectric Microwave Irradiation and a SrO Catalyst. Bioresour. Technol..

[11] Riechmann J.L., Heard J., Martin G., Reuber L., Jiang C., Keddie J., Adam L., Pineda O., Ratcliffe O.J., Samaha R.R. (2000). Arabidopsis Transcription Factors: Genome-Wide Comparative Analysis among Eukaryotes. Science.

[12] Zhu J.-K. (2016). Abiotic Stress Signaling and Responses in Plants. Cell.

[13] Li J., Tian J., Zhou M., Tian J., Liang C. (2025). Research Progress on the Physiological and Molecular Mechanisms Underlying Soybean Aluminum Resistance. New Crops.

[14] Li C., Chen Y., Hu Q., Yang X., Zhao Y., Lin Y., Yuan J., Gu J., Li Y., He J. (2024). PSEUDO-RESPONSE REGULATOR 3b and Transcription Factor ABF3 Modulate Abscisic Acid-Dependent Drought Stress Response in Soybean. Plant Physiol..

[15] Zhu M., Li Y., Wang L., Zhang W., Niu L., Hu T. (2023). Unraveling Antibiotic Resistomes Associated with Bacterial and Viral Communities in Intertidal Mudflat Aquaculture Area. J. Hazard. Mater.

[16] Zhang Y., Han X., Su D., Liu C., Chen Q., Qi Z. (2023). An Analysis of Differentially Expressed and Differentially m6A-Modified Transcripts in Soybean Roots Treated with Lead. J. Hazard. Mater..

[17] Zhang L., Zhang Y., Liu J., Li H., Liu B., Zhao T. (2023). N6-Methyladenosine mRNA Methylation Is Important for the Light Response in Soybean. Front. Plant Sci..

[18] Liu P., Liu H., Zhao J., Yang T., Guo S., Chang L., Xiao T., Xu A., Liu X., Zhu C. (2024). Genome-Wide Identification and Functional Analysis of mRNA m^6^A Writers in Soybean under Abiotic Stress. Front. Plant Sci..

[19] Tang J., Yang J., Lu Q., Tang Q., Chen S., Jia G. (2022). The RNA N6 -Methyladenosine Demethylase ALKBH9B Modulates ABA Responses in Arabidopsis. J. Integr. Plant Biol..

[20] Liang Z., Riaz A., Chachar S., Ding Y., Du H., Gu X. (2020). Epigenetic Modifications of mRNA and DNA in Plants. Mol. Plant.

[21] Wei S., Zhang W., Fu R., Zhang Y. (2021). Genome-Wide Characterization of 2-Oxoglutarate and Fe(II)-Dependent Dioxygenase Family Genes in Tomato during Growth Cycle and Their Roles in Metabolism. BMC Genom..

[22] Cannon S.B., Mitra A., Baumgarten A., Young N.D., May G. (2004). The Roles of Segmental and Tandem Gene Duplication in the Evolution of Large Gene Families in Arabidopsis Thaliana. BMC Plant Biol..

[23] Huang W., Hu X., Ren Y., Song M., Ma C., Miao Z. (2023). IPOP: An Integrative Plant Multi-Omics Platform for Cross-Species Comparison and Evolutionary Study. Mol. Biol. Evol..

[24] Cheng Y., Wang X., Cao L., Ji J., Liu T., Duan K. (2021). Highly Efficient Agrobacterium Rhizogenes-Mediated Hairy Root Transformation for Gene Functional and Gene Editing Analysis in Soybean. Plant Methods.

[25] Wang T., Guo J., Peng Y., Lyu X., Liu B., Sun S., Wang X. (2021). Light-Induced Mobile Factors from Shoots Regulate Rhizobium-Triggered Soybean Root Nodulation. Science.

[26] Kereszt A., Li D., Indrasumunar A., Nguyen C.D., Nontachaiyapoom S., Kinkema M., Gresshoff P.M. (2007). Agrobacterium Rhizogenes-Mediated Transformation of Soybean to Study Root Biology. Nat. Protoc..

[27] Fan Y., Zhang X., Zhong L., Wang X., Jin L., Lyu S. (2020). One-Step Generation of Composite Soybean Plants with Transgenic Roots by *Agrobacterium rhizogenes*-Mediated Transformation. BMC Plant Biol..

[28] Zhao Y., Guo Q., Cao S., Tian Y., Han K., Sun Y., Li J., Yang Q., Ji Q., Sederoff R. (2022). Genome-Wide Identification of the *AlkB* Homologs Gene Family, PagALKBH9B and PagALKBH10B Regulated Salt Stress Response in Populus. Front. Plant Sci..

[29] Han R., Shoaib Y., Cai J., Kang H. (2023). ALKBH10B-Mediated m^6^A Demethylation Is Crucial for Drought Tolerance by Affecting mRNA Stability in *Arabidopsis*. Environ. Exp. Bot..

[30] Tang J., Yang J., Duan H., Jia G. (2021). ALKBH10B, an mRNA m^6^A Demethylase, Modulates ABA Response During Seed Germination in Arabidopsis. Front. Plant Sci..

[31] Shen H., Luo B., Wang Y., Li J., Hu Z., Xie Q., Wu T., Chen G. (2022). Genome-Wide Identification, Classification and Expression Analysis of m^6^A Gene Family in Solanum Lycopersicum. Int. J. Mol. Sci..

[32] Shao L., Xing F., Xu C., Zhang Q., Che J., Wang X., Song J., Li X., Xiao J., Chen L.-L. (2019). Patterns of Genome-Wide Allele-Specific Expression in Hybrid Rice and the Implications on the Genetic Basis of Heterosis. Proc. Natl. Acad. Sci. USA.

[33] Chen K., Li G.-J., Bressan R.A., Song C.-P., Zhu J.-K., Zhao Y. (2020). Abscisic Acid Dynamics, Signaling, and Functions in Plants. J. Integr. Plant Biol..

[34] Wang S., Wang H., Xu Z., Jiang S., Shi Y., Xie H., Wang S., Hua J., Wu Y. (2023). m^6^A mRNA Modification Promotes Chilling Tolerance and Modulates Gene Translation Efficiency in Arabidopsis. Plant Physiol..

[35] Chen D., Fu L., Su T., Xiong J., Chen Y., Shen Q., Kuang L., Wu D. (2022). N6-Methyladenosine Methylation Analysis Reveals Transcriptome-Wide Expression Response to Salt Stress in Rice Roots. Environ. Exp. Bot..

[36] Hou N., Li C., He J., Liu Y., Yu S., Malnoy M., Mobeen Tahir M., Xu L., Ma F., Guan Q. (2022). MdMTA-mediated m^6^A Modification Enhances Drought Tolerance by Promoting mRNA Stability and Translation Efficiency of Genes Involved in Lignin Deposition and Oxidative Stress. New Phytol..

[37] Tang J., Chen S., Jia G. (2023). Detection, Regulation, and Functions of RNA N6-Methyladenosine Modification in Plants. Plant Commun..

[38] Shen H., Zhou Y., Liao C., Xie Q., Chen G., Hu Z., Wu T. (2023). The AlkB Homolog SlALKBH10B Negatively Affects Drought and Salt Tolerance in Solanum Lycopersicum. IJMS.

[39] Tamura K., Stecher G., Kumar S. (2021). MEGA11: Molecular Evolutionary Genetics Analysis Version 11. Mol. Biol. Evol..

[40] Xie J., Chen Y., Cai G., Cai R., Hu Z., Wang H. (2023). Tree Visualization By One Table (tvBOT): A Web Application for Visualizing, Modifying and Annotating Phylogenetic Trees. Nucleic Acids Res..

[41] Chen C., Wu Y., Li J., Wang X., Zeng Z., Xu J., Liu Y., Feng J., Chen H., He Y. (2023). TBtools-II: A “One for All, All for One” Bioinformatics Platform for Biological Big-Data Mining. Mol. Plant.

[42] Anderson S.J., Kramer M.C., Gosai S.J., Yu X., Vandivier L.E., Nelson A.D.L., Anderson Z.D., Beilstein M.A., Fray R.G., Lyons E. (2018). N6-Methyladenosine Inhibits Local Ribonucleolytic Cleavage to Stabilize mRNAs in *Arabidopsis*. Cell Rep..

[43] Wang W., Liu H., Wang F., Liu X., Sun Y., Zhao J., Zhu C., Gan L., Yu J., Witte C.-P. (2023). N4-Acetylation of Cytidine in mRNA Plays Essential Roles in Plants. Plant Cell.

[44] Gao S., Sun Y., Chen X., Zhu C., Liu X., Wang W., Gan L., Lu Y., Schaarschmidt F., Herde M. (2023). Pyrimidine Catabolism Is Required to Prevent the Accumulation of 5-Methyluridine in RNA. Nucleic Acids Res..

[45] Livak K.J., Schmittgen T.D. (2001). Analysis of Relative Gene Expression Data Using Real-Time Quantitative PCR and the 2^−ΔΔCT^ Method. Methods.

[46] Chen M., Witte C.-P. (2020). A Kinase and a Glycosylase Catabolize Pseudouridine in the Peroxisome to Prevent Toxic Pseudouridine Monophosphate Accumulation. Plant Cell.

[47] Martin K., Kopperud K., Chakrabarty R., Banerjee R., Brooks R., Goodin M.M. (2009). Transient Expression in Nicotiana Benthamiana Fluorescent Marker Lines Provides Enhanced Definition of Protein Localization, Movement and Interactions in Planta. Plant J..

[48] Liu J., Tan M., Zhang Y., Zhao J., Liu H., Liu P., Meng W., Ding A., Xiang Z., Chen M. (2024). Dissecting the Roles of Increased mRNA m^6^A Methylation in Autotetraploidization in *Stevia Rebaudiana*. Plant Growth Regul..

[49] Zhu C., Liu X., Wang W., Chen X., Gao S., Qian M., Yang N., Xu Y., Chen M. (2021). Plant Sample Preparation for Nucleoside/Nucleotide Content Measurement with An HPLC-MS/MS. J. Vis. Exp..

