# Supplementary material for: Genome-Wide Identification of the Soybean AlkB Homologue Gene Family and Functional Characterization of GmALKBH10Bs as RNA m6A Demethylases and Expression Patterns under Abiotic Stress
Source: Plants (Basel). 2024 Sep 5;13(17):2491. doi: 10.3390/plants13172491 (PMC11397283; doi:10.3390/plants13172491)
Supplement: Supplementary file 1 [file plants-13-02491-s001.zip › Supplemental Figure S2.pdf]

```
GmALKBH10B1    1 MAAGPTTTSPSDRPTMVPPPMLVSDSFAKDAILAWFRGEFAAANAIIDALCAHLS---ASSAH  60
GmALKBH10B3    1 --MAAGPTSLSDRSTMVPPPMLVSDSFAKDAILAWFRGEFAAANAIIDALCAHLSSAAASSAH  61
GmALKBH10B2    1 MAAVPASRTDPPPAMVAPPPLLVSDSFAKDAILAWFRGEFAAANAIIDSLCGHLAH-LAAASS  62
GmALKBH10B4      ---------------------------------------------------------------

GmALKBH10B1   61 DYDAVFTAIHRRRLNWIPVLQMQKYHSIADVTLELARLADRN----HNSAA----AEHETDDK 115
GmALKBH10B3   62 DYDAVFAAIHRRRLNWIPVLQMQKYHSIADVTLELARLADRN----HYAAAEDDDAKHETDEK 120
GmALKBH10B2   63 DYDATFTAIHRRRLNWIPVIQMQKYHSIADVTLELRRVAEKKTETEAAKKSESSFDEEGKLEK 125
GmALKBH10B4      ---------------------------------------------------------------

GmALKBH10B1  116 TTPSESVGNGGGGDEHEEYESPESEITDSGSQEMQASPMNVNICSNHEECEGRSSQFKLTKGF 178
GmALKBH10B3  121 TTPSESVGDGGGGDEHEEYESPESEITDSGSQEMQASPTNVNICSNHEQCEGRSSQFKLTKGF 183
GmALKBH10B2  126 QAVENGGNDGGDDDAAPVYDSPDSEITDSGSQEMQPNVMNTNICSNHEECEGRSSQIKLTKGF 188
GmALKBH10B4    1 ---------------------------------MQPSVMNNNICSNHEECEGRSSQIKLTKGF  30

GmALKBH10B1  179 AAKESVKGHMVNVVKGLKLYEDIFTDSELCKLTDFVNEIHAAGQNGELSGETFILFNKQMKGN 241
GmALKBH10B3  184 TAKESVKGHMENVVKGLKLYEDIFTDSELCKLTDFVNEIHAAGQNGELSGETFILFNKQMKGN 246
GmALKBH10B2  189 TAKESVKGHMVNVVKGLKLYEDVFSESEICKLTDFVNEIHAAGQNGELSGETFILFNKQMKGN 251
GmALKBH10B4   31 TAKESVKGHMVNVVKGLKLYEDVFSESEICKLTDFVKEIHAAAQNGELSGETFILFNKQMKGN  93

GmALKBH10B1  242 KRELIQLGVPIFGQIKEDAKSNIEPIPVLLQGVIDHLIQWQLLPEYKRPNGCIINFFEKGEFS 304
GmALKBH10B3  247 KRELIQLGVPIFGQIKEDAKCNIEPIPALLQGVIDHLIQWQLLPEYKRPNGCIINFFEEGEFS 309
GmALKBH10B2  252 KRELIQLGVPIFGQIKDDTKNNIEPIPALLHDVIDHLIQWKLIPEYKRPNGCIINFFEEEEFS 314
GmALKBH10B4   94 KRELIQLGVPIFRQIKDDNKSNIEPIPALLHDVIDHLIQWKLIPEYKRPNGCIINFFEEGEFS 156

GmALKBH10B1  305 QPFLKPPHLDQPVSTLLLSESAMAFGRILMSENDGNYKGPLTLSLKQGSLLVMRGNSADMARH 367
GmALKBH10B3  310 QPFLKPPHLDQPVSTLLLSESTMAFGRILMSENDGNYKGPLTLSLKQGSLLVMRGNSADMARH 372
GmALKBH10B2  315 QPFLKPPHLDQPLSTLLLSESTMAFGRILTSENDGNYKGPLMLSLKEGSLLVMRGNSADMARY 377
GmALKBH10B4  157 QPFLKPPHLDQPLSTLLLSESTMAFGRILMSENDGNYKGPLMLSLKEGSLLVMRGNSADMARH 219

GmALKBH10B1  368 VMCPSPNRRVSITFFRVRPDSNQCQTPTPTTMTSAMTMWQPGIAASPYALPNSALTSYEGMDM 430
GmALKBH10B3  373 VMCPSPNRRVSITFFRVRPDSNQCQSPTPTTMTSAMTVWQPGIAASPYALPNGALTSYEGMDM 435
GmALKBH10B2  378 VMCPSPNRRVSITFFRVRPDSNHCQSPTP-TMTTAMTLWHP-SISSPFTLPKGPLNGYEAMDM 438
GmALKBH10B4  220 VMCPSPNRRVSITFFRVRPDSNQCQSPTP-TMTTAMTLWQP-SIASPFTLPNGPLSGYEAMNM 280

GmALKBH10B1  431 NMMPKWGMLHAPMVMLTPMRPVALNPRKLAGGGTGVFLPWNVPSRKPAKHLPPRAQKGRLLTL 493
GmALKBH10B3  436 NMMPKWGMLRAPMVMLTPMRPVALNPHKLSGGGTGVFLPWNVPSRKPAKHLPPRAQKGRLLTL 498
GmALKBH10B2  439 --MPQWGLLSAPMVMLTPMRPMAVNTRKLPRGGTGVFLPWKGASRKHTRHLPPRAQKGRLMEL 499
GmALKBH10B4  281 --MPQLGILSAPMVMLAPMRPMAANTHKLPRGGTGVFLPWKGSARKHARHLPPRAQKGRLMEL 341

GmALKBH10B1  494 PSSVEPQMGESTSEPSICVEG 514
GmALKBH10B3  499 PSPVEPQMGESTS-------- 511
GmALKBH10B2  500 PSPVESHMGESTSEPSIAVEG 520
GmALKBH10B4  342 PSPVESHTGESISEPSIAVEG 362
```
