# Supplementary material for: Genome-Wide Identification of the Soybean AlkB Homologue Gene Family and Functional Characterization of GmALKBH10Bs as RNA m6A Demethylases and Expression Patterns under Abiotic Stress
Source: Plants (Basel). 2024 Sep 5;13(17):2491. doi: 10.3390/plants13172491 (PMC11397283; doi:10.3390/plants13172491)
Supplement: Supplementary file 1 [file plants-13-02491-s001.zip › Supplemental Table S1.pdf]

Supplemental Table S1. Predicted *ALKBH* family genes from *Arabidopsis thaliana* and *Glycine max*

Family	Species	Gene ID	numbers
ALKBH1	*Arabidopsis thaliana*	AT1G11780<br>AT3G14140<br>AT3G14160<br>AT5G01780	4
	Glycine max	Glyma.11G250800<br>Glyma.18G006200<br>Glyma.19G263000<br>Glyma.20G056000<br>Glyma.01G129600	5
ALKBH2	*Arabidopsis thaliana*	AT2G22260	1
	Glycine max	Glyma.09G014800<br>Glyma.15G120500	2
ALKBH6	*Arabidopsis thaliana*	AT4G20350	1
	Glycine max	Glyma.09G156400<br>Glyma.16G207100	2
ALKBH7	*Arabidopsis thaliana*	AT4G02485	1
	Glycine max	Glyma.14G026500	1
ALKBH8	*Arabidopsis thaliana*	AT1G31600	1
	Glycine max	Glyma.09G217100	1
ALKBH9	*Arabidopsis thaliana*	AT1G48980<br>AT2G17970<br>AT4G36090	3
	Glycine max	Glyma.08G186500<br>Glyma.14G106000	2
ALKBH10	*Arabidopsis thaliana*	AT2G48080<br>AT4G02940<br>AT1G14710	3
	Glycine max	Glyma.02G149900<br>Glyma.03G149900<br>Glyma.10G023900<br>Glyma.19G152900<br>Glyma.05G138600<br>Glyma.07G175300<br>Glyma.08G093800<br>Glyma.20G012100<br>Glyma.02G149900<br>Glyma.03G149900<br>Glyma.10G023900<br>Glyma.18G195500	12
