# Supplementary material for: Genome-Wide Identification of the Soybean AlkB Homologue Gene Family and Functional Characterization of GmALKBH10Bs as RNA m6A Demethylases and Expression Patterns under Abiotic Stress
Source: Plants (Basel). 2024 Sep 5;13(17):2491. doi: 10.3390/plants13172491 (PMC11397283; doi:10.3390/plants13172491)
Supplement: Supplementary file 1 [file plants-13-02491-s001.zip › Supplemental Table S2.pdf]

Supplemental Table S2. Predicted conserved motif within the *GmALKBH* family genes in *G. max*.

	Amino Acid Sequence
Motif1	CIIBFFEEGEFSQPFLKPPHLGRPVYTLLLSESEMAFGRIJMSEHPGNYK
Motif2	KGFIAKESVDGKMVNVVKGLKLYEDIFSATEICKLVDFVNEJRAAGKKGZ
Motif3	GPITLSLKPGSLLVMRGNSADMAKHALPSVPNRRISITFTRVRP
Motif4	VGEPGEYDMVVGAIQQRRCNWIQVLQMQQYFSVADVTLALQQVAWRRQQR
Motif5	ZTFIVSNKPMKGNGRELIQLGVPIADAPK
Motif6	DKNVEPIPALFQDIIDRLIQWQVLPEKPR
Motif7	RDGIJGWLRSEFAAANAIIDSLCHHLR
Motif8	PDSCIVNIYEEGDGJGPHQDNHEFVDSLA
Motif9	PMVMLTPMRPPAHPPPKLPAPGTGVFLPP
Motif10	HWGPPPSRSPNHVRHQLGPKHYPTLPATGVLPAPPIRPPM
