# Supplementary material for: Genome-Wide Identification of the Soybean AlkB Homologue Gene Family and Functional Characterization of GmALKBH10Bs as RNA m6A Demethylases and Expression Patterns under Abiotic Stress
Source: Plants (Basel). 2024 Sep 5;13(17):2491. doi: 10.3390/plants13172491 (PMC11397283; doi:10.3390/plants13172491)
Supplement: Supplementary file 1 [file plants-13-02491-s001.zip › Supplemental Table S3.pdf]

Primers used in this study.

Primers used for gene expression analysis (quantitative real-time PCR)

Primer number	Sequence (5'-3')	Gene ID	Production length
Cp363	gaatcggtgggagatttagcg	*Glyma.12G051100*	120bp
Cp364	ccaaagagtgtcatcggaag	*Glyma.12G051100*	120bp
Cp639	ggaagggaggcctcgagtgtacgaa	*Glyma.19G152900*	125bp
Cp640	ttgagtgagtcacatgagatgcag	*Glyma.19G152900*	125bp
Cp641	cgtaaaccctttctcaaagcacag	*Glyma.02G149900*	151bp
Cp642	atcggagggtgatgttgttgtaggg	*Glyma.02G149900*	151bp
Cp643	cgggcacagggattgttagaagag	*Glyma.03G149900*	168bp
Cp644	ccaaatttggaagcaagacttctcc	*Glyma.03G149900*	168bp
Cp645	ccgatcactgcgtatcatcacaatt	*Glyma.10G023900*	104bp
Cp646	gcgaaggagtcggagacgagcatt	*Glyma.10G023900*	104bp

Primers used for plasmid construction

Primer number	Sequence (5'-3')	Gene ID	Production length
Cp996	gatatcgaattcctgcagcccatggcggctggccctaca	*Glyma.02G149900/ Glyma.10G023900*	1542bp/ 1533bp
Cp997	ctcgcccttgctcaccatccctccttcaacagcaatgct	*Glyma.03G149900*	1560bp
Cp998	ctcgcccttgctcaccatccctccttcaacacaaatgct	*Glyma.02G149900/ Glyma.19G152900*	1545bp/ 1086bp
Cp999	gatatcgaattcctgcagcccatggcggctgtcccagca	*Glyma.03G149900*	1560bp
Cp1000	ctcgcccttgctcaccatcccagaagtggactctcccat	*Glyma.10G023900*	1533bp
Cp1001	gatatcgaattcctgcagcccatgcaacccagtgtgatg	*Glyma.19G152900*	1086bp

The red sequences represent the homology arms.
