# Supplementary figures and images for: Genome-Wide Identification of the Soybean AlkB Homologue Gene Family and Functional Characterization of GmALKBH10Bs as RNA m6A Demethylases and Expression Patterns under Abiotic Stress
Source: Plants (Basel). 2024 Sep 5;13(17):2491. doi: 10.3390/plants13172491 (PMC11397283; doi:10.3390/plants13172491)

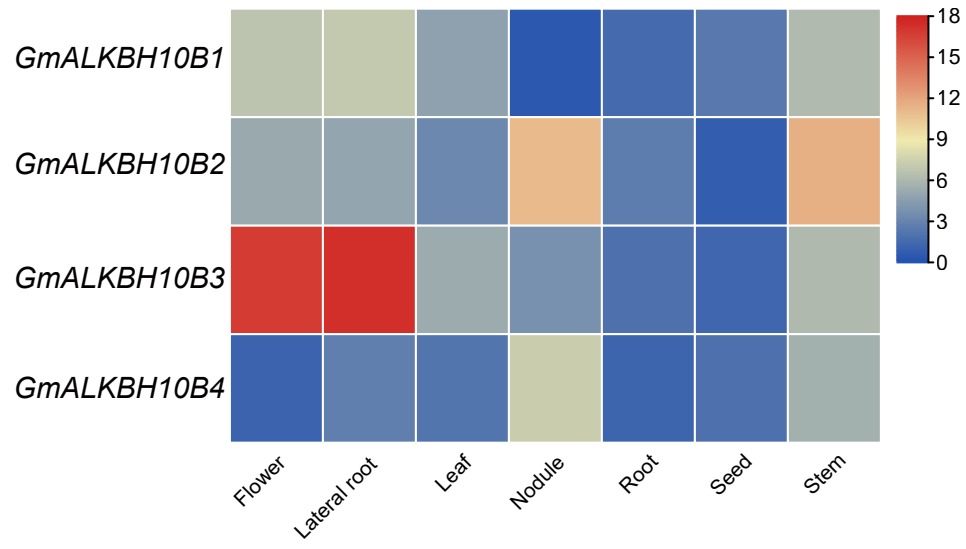

Supplement: Supplementary file 1 [file plants-13-02491-s001.zip › Supplemental Figure S1.pdf]
